# Medical education system in Malaysia maintaining a gold standard - an extraordinary example from the Malaysian Society of Pharmacology and Physiology

**DOI:** 10.3126/nje.v7i3.19008

**Published:** 2017-09-30

**Authors:** Bedanta Roy, Nicholas Goh, Rukhsana Hussain Malik

**Affiliations:** 1 Senior Lecturer, Department of Physiology, Faculty of Medicine Quest International University Perak (QIUP) City Campus, No.227, Plaza Teh Teng Seng (Level 2), Jalan Raja Permaisuri Bainun, 30250 Ipoh, Perak Darul Ridzuan, Malaysia.; 2 Chief Operating Officer, Quest International University Perak (QIUP) City Campus, No.227 Plaza Teh Teng Seng (Level 2), Jalan Raja Permaisuri Bainun, 30250 Ipoh, Perak Darul Ridzuan, Malaysia.; 3 Professor and Head, Medical Education, Faculty of Medicine Quest International University Perak (QIUP) City Campus, No.227, Plaza Teh Teng Seng (Level 2), Jalan Raja Permaisuri Bainun, 30250 Ipoh, Perak Darul Ridzuan, Malaysia

Sir,

"Competition is always a good thing. It forces us to do our best. A monopoly renders people complacent and satisfied with mediocrity "a quote by Nancy Pearcey, an American evangelical author. Malaysian Society of Pharmacology and Physiology (MSPP) invites teachers of Physiology and Pharmacology (should be below 45 years of age) from different universities in Malaysia to participate in Teacher 's Prize competition, with one faculty being awarded in the respective fields each year. A selected panel of senior professors from prestigious Institutions across the country evaluates the presentation and selects the best presenter. Teacher 's Prize 2017 in Physiology was held at Universiti Teknologi MARA (UiTM), Sungai Buloh Campus, Selangor, Malaysia. There was a tough challenge amongst the academic staff that came from renowned public and private universities. Participants used teaching aids like Power Point presentations, animations, short video clips etc. to add flavor to their teaching. The prize was awarded to QIUP lecturer, Faculty of medicine at the closing ceremony of the 31st Scientific Meeting of MSPP in Universiti Sains Malaysia, Kota Bharu, Kelantan.

Currently, there are 32 medical schools in Malaysia (11 public and 21 private) [ 1 ]. Apart from Malaysian faculties, experienced expatriates from India, Pakistan, Bangladesh, Myanmar and Indonesia are also teaching in medical fields in various Malaysian Universities. A teacher 's understanding on the subject and his method of presentation is one main thing which creates an enthusiasm or a spark in the students. We should also keep in mind that we teach should be how we learnt so that it makes both an easy task. Also, a balance should be maintained on the topic taught per day else would result inburdening the students. 

 Founded in 2011 Quest International University Perak (QIUP) is a private university in partnership with Perak state Government. Various professional programmes are conducted in Faculty of Medicine (FOM) helps the young lecturers. Enormous financial aids and moral support are provided by the University for competitive events which are highly appreciable. Universities across the world should encourage their faculties, to organize and participate healthy competitions like quiz, teachers ' prize to make a better educator who guides future health care professionals.

Dr. Bedanta Roy, Senior Lecturer, Department of Physiology, Faculty of Medicine, Quest International University Perak (QIUP) City Campus, No.227, Plaza Teh Teng Seng (Level 2), Jalan Raja Permaisuri Bainun, 30250 Ipoh, Perak Darul Ridzuan, Malaysia. 

 Mr. Nicholas Goh, Chief Operating Officer, Quest International University Perak (QIUP) City Campus, No.227, Plaza Teh Teng Seng (Level 2), Jalan Raja Permaisuri Bainun, 30250 Ipoh, Perak Darul Ridzuan, Malaysia. 

 Prof. Dr. Rukhsana Hussain Malik, Professor and Head, Medical Education, Faculty of Medicine, Quest International University Perak (QIUP) City Campus, No.227, Plaza Teh Teng Seng (Level 2), Jalan Raja Permaisuri Bainun, 30250 Ipoh, Perak Darul Ridzuan, Malaysia. 

 Paediatrics, QIUP; Professor Dr. Subhada Prasad Pani, Deputy Dean (Pre-Clinical), Faculty of Medicine and Research Professor and Head, Department of Microbiology, QIUP; Prof. Dr. Sumitabha Ghosh, Professor and Head of Department, Physiology, Faculty of Medicine, QIUP and all the faculty members in FOM, QIUP for their support and encouragement. 

 Dated the 22nd September 2017
